# Special Issue: Recent Advances in Corrosion Science

**DOI:** 10.3390/ma13081927

**Published:** 2020-04-19

**Authors:** Jacek Ryl

**Affiliations:** Department of Electrochemistry, Corrosion and Materials Engineering, Gdansk University of Technology, Narutowicza 11/12, 80-233 Gdansk, Poland; jacek.ryl@pg.edu.pl

The International Union of Pure and Applied Chemistry (IUPAC) and European Federation of Corrosion (EFC) define corrosion as an irreversible interfacial reaction of a material with its environment which results in its consumption or dissolution, often resulting in effects detrimental to the usage of the material considered. Corrosion failure is a significant problem in any given type of industry, leading to substantial economic consequences, but also often influencing human health and the environment negatively, among other unmeasurable factors. The industry estimates indicate that the total direct cost of corrosion ranges between 3% and 5% of GDP [1], while the indirect costs (outages, delays, revenue losses, etc.) while much harder to evaluate, are estimated to be equal to this. These numbers point out that investments in corrosion protection are, by all means, economically justified.

The dynamic development of the global industry and growing demand for new material technologies generates constantly increasing problems regarding premature material degradation and the requirement to determine corrosion mechanisms and to develop new protection/evaluation approaches. This Special Issue, “Recent Advances in Corrosion Science”, brings together fourteen articles and one review, providing a snapshot of the recent activity and development in this field.

The corrosion properties of ferrous metals remain the most popular subject of investigation, which naturally found coverage in numerous research articles present within this Special Issue. The primary source of this versatility is achieved by a proper selection of alloying additives and metalworking, which guarantee the demanded mechanical and physicochemical properties. On the other hand, the alteration of metal structure leads to the formation of galvanic microcells, often translating into various forms of local corrosion. The search for alloying additives enhancing the corrosion resistance without sacrificing the desired characteristics continues, intending to reduce alloy corrosion rate and bring measurable economic profits. Within this Special Issue, you will find multiple original research papers strictly devoted to this issue for both ferrous [2,3,4] and non-ferrous metals [5,6,7,8]. The influence of novel microscopy tools, which enable the direct observation of local corrosion processes, cannot be overestimated. For this reason, I would like to recommend a very interesting and important review prepared by Chen et al. [9], referring to the advances in electrochemical atomic force microscopy (EC-AFM), an outstanding tool to perform real-time in situ corrosion studies of galvanic microcells.

Affecting the corrosion process by electrochemical protection (cathodic or anodic), barrier properties obtained with the use of paints or coatings as well as environment modification with dedicated corrosion inhibitors, are the three primary ways to reduce the corrosion rate found in both principle and industrial studies regarding anti-corrosion technologies. All of these research areas are represented within this Special Issue. The works of Xu et al. [10], Tang. et al. [11] and Ryl et al. [12] reveal various aspects concerning the search for efficient organic corrosion inhibitors and the tools used to evaluate protection mechanisms. The studies of Parchoviansky et al. [13] and Winiarski et al. [14] provide an insight on the development of anti-corrosion composite coatings, while an interesting report from Kania and Sipa [15] shows the improved corrosion resistance of anodic zinc coatings, obtained using a new thermal diffusion process.

It is important to emphasize that, nowadays, corrosion issues are not solely connected with the degradation of metals. Modern composite or semiconductor electrode materials are constantly developed to be used in numerous branches of applied electrochemistry, such as energy storage and conversion, electrochemical sensors and electrocatalytic processes. Their stable performance under aggressive environmental factors is often questionable. Thus, the final manuscript of this Special Issue presents work in this new field, which was devoted to high-temperature oxidation and the degradation of boron-doped diamond nanostructures [16]. 
